# Spatio-temporal dynamics and trend forecasting of urban green high-quality development: An examination of 287 cities in China

**DOI:** 10.1371/journal.pone.0320894

**Published:** 2025-04-11

**Authors:** Tonghui Yu, Shanshan Jia, Xufeng Cui

**Affiliations:** 1 School of Business, Xinyang Normal University, Xinyang, Henan, China,; 2 School of Business Administration, Zhongnan University of Economics and Law, Wuhan, Hubei, China; Southwest Jiaotong University, CHINA

## Abstract

Facing the dual challenges of economic growth and environmental conservation, advancing urban green high-quality development (UGHQD) is crucial for sustainable urban development. Rooted in the concept of UGHQD, this study develops a multidimensional evaluation indicator system encompassing economic development, social progress, and ecological civilization. By applying spatial autocorrelation analysis, cold and hot spots analysis, standard deviation ellipse, and Kernel density estimation, it examines the spatial distribution and dynamic evolution of UGHQD across China and its four major regions (Eastern, Central, Western, and Northeastern) from 2003 to 2020. It also forecasts the trajectory of UGHQD from 2021 to 2025. The research findings indicate: (1) A steady annual increase in the overall level of UGHQD, with a geographic pattern showing high levels in the east, moderate in the center, and low in the west. (2) A spatial agglomeration in UGHQD, predominantly in the eastern region, demonstrating spatio-temporal inertia. (3) Varying degrees of a “right-tail” phenomenon in the UGHQD across China and its four sub-regions, indicating a polarization trend or even a weak multi-polarization trend. (4) A forecast of continuous, steady growth in UGHQD from 2021 to 2025, with the eastern region maintaining its leading position. This study offers insights that enhancing our understanding of the fundamental concepts underlying UGHQD, providing a practical foundation and policy guidance for future collaborative efforts in enhancing urban development quality.

## 1. Introduction

As a pivotal actor in the global economy, China has demonstrated remarkable resilience and dynamism, serving as a robust engine of global economic growth and a cornerstone of worldwide economic stability. With its economy progressing in a phase of orderly and accelerated growth, China is increasingly unleashing its innovative potential and development capabilities, exhibiting a continually improving growth trajectory. However, this rapid growth has not been without challenges, notably exacerbated environmental pollution, imbalanced resource utilization, and suboptimal development quality and efficiency [1–3]. The report to the 20th National Congress of the Communist Party of China explicitly states that “high-quality development (HQD) is the primary task of comprehensively building a modern socialist country”, and emphasizes that “promoting the green and low-carbon transition of economic and social development is crucial for achieving HQD”. In this new developmental phase, there is an urgent need to move away from inefficient, pollution-intensive economic models, incorporate sustainable development principles within the wider HQD strategy, and achieve sustainable economic and social progress within environmental carrying capacities [4, 5].

Against this backdrop, balancing environmental conservation with economic growth is crucial, as it simultaneously advances economic development, social progress, and ecological civilization [6, 7]. This approach underpins green, low-carbon, efficient, and high-standard development. Currently, developing a sustainable development paradigm that fosters the positive interactions among ecological, economic, and social elements is essential for sustainable and healthy economic progress. This paradigm serves as a vital foundation for a balanced relationship between humanity and the natural environment, addressing ecological challenges and enhancing development quality [8].

Therefore, this study systematically investigates into the essence, characteristics, and trends of urban green high-quality development (UGHQD) to promote economic, ecological, and social well-being. It aims to foster a greener, healthier, and more harmonious global development model, while offering guidance on sustainable resource utilization and ecological protection. By enhancing UGHQD, the study aims to improve environmental quality management, optimize resource allocation, and support the harmonious coexistence of the economy and ecology. This contributes to achieving the United Nations’ Sustainable Development Goals, particularly enhancing ecosystem health and biodiversity, thus laying a solid foundation for global sustainability.

HQD delineates the long-term economic trajectory envisioned for contemporary China and has emerged as a focal point in recent scholarly discourse. Theoretical exploration in this field has significantly deepened, generating diverse and multidimensional research perspectives [9, 10]. Some studies adopt economic development as the lens through which to analyze HQD [11, 12], underscoring the necessity for China’s economy to transition toward a path of healthy and stable growth [13, 14]. This shift necessitates a move away from traditional factor-dependent growth models toward a greater focus on technological innovation and ecological sustainability, fostering a balanced relationship between human societies and the natural environment [15, 16]. Grounded in the five development concepts of innovation, coordination, green, openness, and sharing, these studies delve into the essence of HQD [17], advocating a shift from “single high-speed growth” to “balanced and stable development”. They promote the adoption of a “people share” model rather than a “being the first to get rich” approach and a reorientation of the development paradigm from a sole focus on GDP to a greater emphasis on “green environmental protection”, aiming to fulfill the long-term objectives of HQD [18, 19]. Building upon a thorough understanding of the essence and characteristics of HQD, methodologies like total factor productivity and comprehensive evaluation indicator systems are employed to delineate this concept [20–23]. Additionally, techniques including data envelopment analysis (DEA), principal component analysis, and entropy-weighted TOPSIS are utilized for calculating the weights of indicators, thereby facilitating a more precise and objective assessment of HQD [24–26]. Furthermore, numerous studies leverage tools such as the Moran index, hot and cold spot analysis, kernel density estimation, and the BP neural network prediction model to uncover the spatial distribution and dynamic progression patterns of HQD levels and to forecast their future trends [27–31]. These insights provide vital theoretical foundations for formulating differentiated and adaptive HQD policies.

UGHQD embodies a holistic development model that prioritizes ecological sustainability while synergizing economic, social, and environmental dimensions. UGHQD’s theoretical exploration dates back to the 1980s, coinciding with the introduction of the sustainable development paradigm, and its profound connotations have been extensively refined through decades of exploration and practice [32]. In examining the interrelationship between sustainability and development, it becomes evident that sustainability serves as the fundamental life support system for development [33, 34]. In 2011, the OECD defined green growth within the framework of sustainable development [35]. Recent research systematically investigates green growth as a transformative economic paradigm fostering circularity, sustainability, and resilient development. Its core principle revolves around “ecological priority and green development”, which aims to foster a synergistic advancement of economic, social, and environmental domains, safeguarding natural resources, and enhancing human well-being [36, 37]. In 2015, China first introduced the pivotal concept of green development, integrating it with innovation, coordination, openness, and sharing to forge a new development paradigm aligned with national conditions, highlighting green as a crucial driving force for benign development. Based on a review of related research, it is evident that UGHQD is not only a continuation of the “lucid waters and lush mountains are invaluable assets” theory but also an economic development model and a path of social progress that seamlessly integrates the imperatives of efficiency and sustainability [38]. Within this context, the UGHQD framework further mandates green principle internalization across productive systems and societal practices to equilibrate economic-ecological dynamics [39].

Quantifying UGHQD stems from green development analytics [40]. In quantitative studies on green development, methodologies measuring efficiency from an input-output viewpoint are extensively used. To capture more comprehensive and systematic information, some studies have constructed evaluation indicator systems for the level of green development and obtained objective results using methods such as entropy weighting and principal component analysis [41–44]. Most studies reveal Chinese urban green development exhibits an upward, “W”-shaped fluctuation pattern [45, 46], with ecological conditions improving alongside sustained progress in eco-conscious development [47]. In recent years, green total factor productivity (GTFP) has been widely employed as a proxy measure for UGHQD attainment [48–50]. Many studies have quantified UGHQD levels by integrating the GTFP of environmental factors, highlighting the mechanisms of interaction and spatial spillover effects between the digital economy and UGHQD [51, 52]. Nevertheless, as UGHQD encompasses economic, social, and ecological dimensions, existing studies lack an integrated framework to comprehensively assess UGHQD levels and their complex connotations.

In summary, the current research on UGHQD has achieved certain results, laying a solid foundation for this study, but there is still urgent research potential to be explored. In measuring UGHQD levels, most studies have relied on GTFP as a proxy variable. However, this method does not provide a comprehensive assessment of a country or region’s overall performance in achieving UGHQD. Furthermore, while many studies have focused on national, provincial, or river basin scales, urban-level research remains limited. Additionally, the current literature largely examines the spatial and temporal dynamics of green or high-quality development, yet it falls short in predicting future trends and identifying potential challenges of UGHQD through predictive models. This study offers the following unique contributions: Firstly, it develops a comprehensive evaluation index system that encompasses economic, social, and ecological dimensions, based on a well-defined UGHQD framework. This system carefully considers the interplay and impact of various indicators, thereby enhancing the precision of our understanding of UGHQD. Secondly, it selects cities as the unit of analysis to more intricately and accurately determine the patterns of UGHQD. Finally, it utilizes a BP neural network model to forecast future trends in UGHQD, elucidating potential developmental challenges and providing robust scientific support for crafting targeted policies.

Focusing on UGHQD, this study establishes a comprehensive evaluation index system that incorporates the dimensions of economic development, social progress, and ecological civilization. It rigorously defines the essential features of UGHQD and employs a variety of research methodologies, including the entropy method, Kernel density analysis, and BP neural network prediction models. These tools assess UGHQD levels across 287 Chinese cities, revealing the spatial distribution patterns and dynamic evolution across the country and its four major regions. The study forecasts future trends and identifies UGHQD’s characteristics, clarifying the mechanisms driving its development. This provides a scientific basis and practical guidance for fostering the coordinated advancement of regional economic prosperity, social stability, and ecological civilization. Moreover, promoting UGHQD is identified as not only a pressing need for China but also a critical direction for sustainable global development.

## 2. Connotation and indicator system of UGHQD

### 2.1 Connotation

This study focuses on the urban level because cities play a pivotal role in modern societies: they serve as engines of economic prosperity, pillars of social stability, and hubs for ecological and environmental protection. This multifunctional positioning makes UGHQD implementation essential for advancing regional sustainability transitions. Emerging from China’s strategic economic restructuring period, the UGHQD concept synthesizes core tenets of green development with HQD imperatives. As a comprehensive development mode that integrates economic development, social progress, and ecological civilization into an organically unified whole, UGHQD aims to achieve a coordinated mechanism for the “trinity” of economy, society, and ecology, thereby attaining an optimal state of development [18, 51].

Initially, UGHQD represents a comprehensive development mode grounded in economic development. From an economic development perspective, fostering a virtuous cycle within the natural ecosystem’s carrying capacity, it is imperative to concentrate on unleashing economic potential while transitioning to and exploring an economic development mode that is not reliant on natural resources. This model emphasizes “enhancing quality and increasing efficiency”, and operates through strategies such as industrial transformation and upgrading, economic structural adjustment, resource allocation optimization, and green innovation fostering. By emphasizing efficiency-driven growth, it accelerates eco-economic synergies to fortify urban resilience.

Secondly, UGHQD represents a benign development approach fundamentally anchored in social stability. In terms of social progress, fulfilling the public’s essential desires for achievement, happiness, and security is imperative. This calls for collaborative efforts and equitable resource sharing among all stakeholders, with a primary focus on enhancing social welfare. By prioritizing social equity and inclusiveness, this approach is dedicated to improving citizens’ quality of life. It involves accelerating the simultaneous advancement of material well-being and spiritual civilization, cultivating a “new mainstream” in green modes of transportation and ecological consumption, and maintaining the dual-engine growth of both the economy and society to continuously expand the gains of UGHQD.

Finally, UGHQD is characterized by a circular development mode underpinned by ecological conservation. From an ecological civilization perspective, guiding economic prosperity and social harmony with a focus on green development is essential. This involves prioritizing green initiatives, energy efficiency, and emission reduction. It is crucial to abandon traditional “high-consumption, high-pollution” modes of production and consumption. Instead, fostering high-quality economic growth through green technological innovation, reducing energy use per unit of GDP, ensuring effective pollution control and environmental governance, and promoting sustainable and recyclable utilization of resources are imperative. Furthermore, establishing an ecological compensation mechanism is vital to forge a more habitable environment, thereby accelerating the transition toward greener urban economies and sustainable social development.

Overall, achieving UGHQD demands an integrated approach that considers economic, social, and environmental dimensions, aiming to strike a harmonious equilibrium among economic growth, social stability, and ecological conservation. By leveraging green innovation and economic restructuring as key levers, it seeks to ensure the sustainability and stability of urban economic development. At this pivotal moment of advancing toward Chinese-style modernization, the emerging paradigm of UGHQD serves as a beacon for building more resilient and diverse cities, guiding us toward a synergistic coexistence between human civilization and the natural environment, while charting a path for an eco-friendly transformation of both the economy and society.

### 2.2 Indicator system.

The existing literature primarily utilizes GTFP as a proxy to assess the level of UGHQD [52, 53]. However, GTFP alone does not suffice for a comprehensive and systematic evaluation of UGHQD. To address this limitation, this study builds upon the connotation of UGHQD, focusing on its core elements: “green” and “high-quality”. It references the research findings of scholars in the realms of sustainable growth and HQD [54–57], adhering to the principles of indicator selection. Consequently, this study develops an evaluation system for UGHQD, which encompasses three dimensions: economic development, social progress, and ecological civilization. The system integrates 29 specific fundamental indicators, as delineated in Table 1.

**Table 1:** Evaluation indicator system for UGHQD.

Guideline layer	Indicator number	Indicator layer	Indicator definition	Unit	Properties	Ref.
**Economic Development**	X_1_	Economic development level	Per capita real GDP	CNY	+	[3,10,20]
	X_2_	Social consumption level	Per capita total retail sales of social consumer goods	CNY	+	[45,58]
	X_3_	Fixed asset investment	Per capita total social fixed asset investment	CNY	+	[30,50]
	X_4_	Average wage level	The average wage of urban workers	CNY	+	[26]
	X_5_	Advancement of industrial structure	Value added of tertiary industry/value added of secondary industry	%	+	[59]
	X_6_	Rationalization of industrial structure	Thiel’s index of structural deviations	–	–	[59]
	X_7_	Science funding input	Per capita science expenditure	CNY	+	[4,58]
	X_8_	Investment in education	Per capita education expenditure	CNY	+	[4,10]
	X_9_	Foreign trade dependence	Total imports and exports/ GDP	%	+	[20]
	X_10_	Foreign investment dependence	Total actual use of foreign capital/ regional GDP	%	+	[4,13]
**Social Progress**	X_11_	Green innovation achievements	Number of green patents granted per 10,000 people	Pieces	+	[4,52]
	X_12_	Internet penetration rate	Internet users per 100 people	household	+	[10]
	X_13_	Transportation infrastructure	Number of road miles per unit of land area	Kilometers	+	[20]
	X_14_	Medical and healthcare level	Number of hospital and health center beds per 10,000 people	Beds	+	[10,13,22]
	X_15_	Basic education conditions	Number of primary and secondary school teachers per student	Peoples	+	[58]
	X_16_	Public library resources	Number of public books owned per 100 people	Books	+	[13,58]
	X_17_	Urban public transportation	Number of vehicles per 10,000 people operating public motor vehicles (electric)	Vehicles	+	[26,58]
	X_18_	Electricity consumption per unit of GDP	Social electricity consumption/GDP	kWh/CNY	–	[13,45]
	X_19_	Water consumption per unit of GDP	Total water supply/GDP	Ton/CNY	–	[45]
	X_20_	Highly educated personnel training	Number of college students per 10,000 people	Peoples	+	[58]
**Ecological Civilization**	X_21_	Annual average air quality	Annual average PM2.5 value	mg/m3	–	[6,10]
	X_22_	Industrial exhaust emissions	Industrial sulfur dioxide emissions/industrial output	Tons/billion	–	[20]
	X_23_	Industrial wastewater discharge	Industrial wastewater discharge/industrial output	Tons/billion	–	[1,3]
	X_24_	Industrial fume emissions	Industrial smoke (dust) emissions/industrial output	Tons/billion	–	[3,36]
	X_25_	Urban greening level	Green coverage of built-up areas	%	+	[22,57]
	X_26_	Green environment construction	Public green space per capita	hectares	+	[1,57]
	X_27_	Solid waste utilization rate	General industrial solid waste comprehensive utilization rate	%	+	[10]
	X_28_	Domestic sewage treatment rate	Centralized treatment rate of sewage treatment plants	%	+	[1,13]
	X_29_	Domestic waste disposal rate	Harmless disposal rate of domestic waste	%	+	[22]

Note: “+ (-)” in the “properties” column indicates that the measure is positive (negative) in the set measurement mode, and the larger (smaller), the better.

#### 2.2.1 Economic development dimension.

Positioning a nation’s or region’s economic development as the groundwork for formulating ecological enhancement strategies is acknowledged as a fundamental prerequisite for advancing UGHQD. This study identifies and evaluates key indicators within the economic development dimension to assess economic development comprehensively. The fundamental indicators of economic development encapsulate a region’s fiscal capacity, potential for scientific and technological innovation, and degree of industrial modernization. These indicators quantify the health and quality of an economy. Specifically, per capita real GDP and per capita total social fixed asset investment measure the economic scale and consumer spending power, reflecting economic prosperity and market vitality. The average wage of urban workers indicates economic welfare levels, serving as a crucial driver of economic development. Investment in science and education fosters technological advancement and talent development, which are pivotal in enhancing economic quality. Dependence on foreign investment and trade gauges a region’s attractiveness to and reliance on international markets and external capital, reflecting the openness of the economy, a critical factor in economic growth. Notably, the modified Thiel index, as refined by Gan et al. [59], is utilized to assess the rationalization of the industrial structure, alongside the value-added ratio of tertiary to secondary industry, indicating the advancement and balance of the economic structure, which plays a significant role in assessing the quality and equilibrium of economic development.

#### 2.2.2 Social progress dimension.

UGHQD requires not only economic development but also a profound emphasis on social progress, stability, and enhancement of living standards. To effectively capture the multidimensional facets of social progress, we have established a set of fundamental indicators. Collectively, these indicators offer a comprehensive depiction of societal progress and are closely aligned with the objectives of UGHQD. These indicators encompass various dimensions including social infrastructure, education, innovation, and resource utilization efficiency, reflecting the level of social development and the quality of life in the region. Specifically, the green innovation and Internet penetration indicators gauge progress in scientific and technological innovation and the application of information technology. Public service and infrastructure indicators, such as transportation infrastructure, healthcare quality, and urban public transportation, assess both the quality of life of urban residents and the efficiency of societal operations. Moreover, indicators of education and cultural resources, such as basic education conditions, public library resources, and higher education talent development, assess the accessibility and quality of educational and cultural offerings, serving as crucial measures of societal and cultural advancement and talent cultivation. The electricity and water consumption per unit of GDP, which gauges resource use efficiency, reflects the society’s commitment to resource management and sustainable development. Collectively, these indicators provide a comprehensive portrayal of societal progress and align closely with the objectives of UGHQD.

#### 2.2.3 Ecological civilization dimension.

Adhering to the principles of ecological civilization and committing to sustainable growth serve as pivotal pathways toward achieving UGHQD. We selected a set of fundamental indicators for the ecological civilization dimension, which encapsulate core aspects such as environmental quality, pollution control, and ecological protection, and effectively demonstrate achievements in ecological civilization. Specifically, the annual average PM2.5 value serves as a crucial indicator for assessing environmental health. Indicators that encompass industrial emissions of exhaust, wastewater, and fumes gauge the impact of industrial activities on the ecological environment. Controlling these emissions is vital for realizing the objectives of ecological civilization. Furthermore, the green coverage of built-up areas and the public green space per capita are significant in enhancing the quality of urban ecological environments, reflecting the city’s efforts and success in fostering ecological civilization. Indicators like the solid waste utilization rate, domestic sewage treatment rate, and domestic waste disposal rate focus on efficient resource use and environmental stewardship, playing a pivotal role in advancing ecological civilization and achieving environmental sustainability. Incorporating these indicators provides a comprehensive depiction of urban environmental governance and outcomes.

## 3. Materials and Methods

### 3.1 Study area

**Fig 1:**
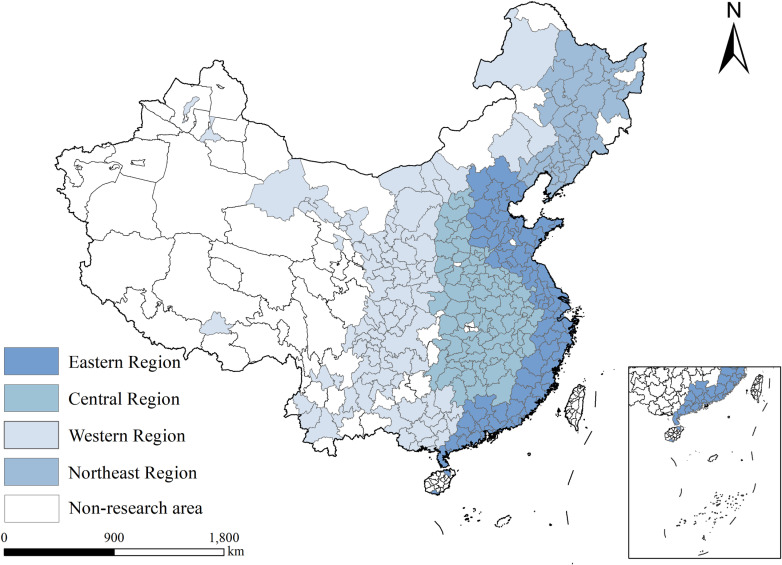
Location of China and four major regions. Note: Quoted from the Tianditu map. Source of base map: the open source map data service provided by the National Platform for Common GeoSpatial Information Services (https://www.tianditu.gov.cn/).

This study examines 287 cities in China, categorized into four major regions—Eastern, Central, Western, and Northeastern—based on geographical and economic characteristics (Fig 1). The Eastern region, known for its advanced economy, strategic location, and high industrial concentration, serves as a key area for studying urban development and green, high-quality growth models in China. The Central region, a focal point of the “Rise of Central China” initiative, is transitioning towards a more diversified economic structure. The Western region, driven by the “Western Development” policy, plays a pivotal role in analyzing the impacts of regional strategies, with significant investments in infrastructure and ecological projects. The Northeastern region, a traditional industrial base, is undergoing industrial transformation and upgrading, offering a unique lens through which to examine economic restructuring and green development. Collectively, these regions represent the principal dimensions of China’s economic and social progress. Therefore, selecting these regions as the research sample facilitates an exploration of common challenges and varied strategies for enhancing UGHQD across different areas.

### 3.2 Methods

#### 3.2.1 Entropy method.

To enhance the scientific validity and accuracy of weight assignment within the indicator system, current research typically employs either subjective or objective weighting methods to address discrepancies in measurement units across constituent indicators. Subjective weighting methods are prone to personal biases, which can result in unreliable conclusions. In contrast, objective weighting methods offer greater universality and reliability. The entropy method, in particular, is favored for its robustness against human bias in indicator weighting, rendering it a prevalent choice for assessing the weights of basic indicators. This study applies the entropy method, building on prior research, to evaluate UGHQD levels from 2003 to 2020. The measurement procedure is as follows:

(1) Acknowledging the potential for varying scales and dimensions within the indicator system, it is imperative to standardize both positive and negative indicators to effectively mitigate their impact on the evaluation outcomes:

$$\text{Positive standardization:}y_{ij}=\frac{x_{ij}-\min(x_{ij})}{\max(x_{ij})-\min(x_{ij})}$$ (1)

$$\text{Negative standardization:}y_{ij}=\frac{\max(x_{ij})-x_{ij}}{\max(x_{ij})-\min(x_{ij})}$$ (2)

Where, *i* represents the year, *j* represents the indicator, *x*_*ij*_ represents the raw data for the indicator, and *y*_*ij*_ represents the normalized value of the indicator.

(2) Calculate the share of the indicator for the *i-th* city under the *j-th* indicator:

$$Y_{ij}=\frac{y_{ij}}{\sum_{i=1}^{m}y_{ij}}$$ (3)

(3) Calculate the information entropy e_j_:

$$e_{j}=-\frac{1}{\ln m}\sum_{i=1}^{m}Y_{ij}\ln Y_{ij},0\leq e_{j}\leq 1$$ (4)

Where, *m* is the total number of evaluation objects, *Y*_*ij*_ represents the normalized data of the indicator after standardization, and *e*_*j*_ is the entropy value of the *j-th* indicator.

(4) Calculate the weight w_j_:

$$w_{j}=\frac{1-e_{j}}{\sum_{j=1}^{n}\left(1-e_{j}\right)}$$ (5)

(5) Each city’s UGHQD score:

$$UGHQD_{i}=\sum_{j=1}^{m}w_{j}\cdot Y_{ij},i=1,2......,m$$ (6)

Where, *UGHQD*_*i*_ is the comprehensive score for the *i-th* evaluation object, *w*_*j*_ is the weight of the *j-th* indicator, and *Y*_*ij*_ is the standardized value of the *i-th* indicator for the *j-th* evaluation object.

#### 3.2.2 Spatial autocorrelation.

Spatial autocorrelation measures the degree of association between the value of a specific attribute on each spatial unit and the same attribute on adjacent spatial units [60]. The most widely used metric for this is Moran’s I, which ranges from -1–1. As Moran’s I approaches 1 or -1, it signifies an increasing strength in the variable’s positive or negative spatial correlation, respectively. Conversely, a Moran’s I value of 0 indicates the absence of spatial correlation between the attributes of neighboring units. In this study, both Global and Local Moran’s I are used to examine the spatial autocorrelation of UGHQD levels across 287 Chinese cities at global and local scales. Global Moran’s I metric quantifies the degree of attribute value correlation across neighboring entities throughout the entire spatial area, with the specific calculation formula outlined as follows:


$$GlobalMoran'sI=\frac{n\sum_{i=1}^{n}\sum_{j=1}^{n}W_{ij}\left(x_{i}-\bar{x}\right)\left(x_{j}-\bar{x}\right)}{(\sum_{i=1}^{n}\sum_{j=1}^{n}W_{ij})\sum_{i=1}^{n}{\left(x_{i}-\bar{x}\right)}^{2}}$$ (7)

Local Moran’s I serves as an indicator for measuring local spatial autocorrelation. This indicator’s value indicates the strength of the association between a spatial entity and the entities adjacent to it. The calculation formula is outlined as follows:


$$LocalMoran'sI=\frac{\left(x_{i}-\bar{x}\right)\sum_{j=1}^{n}W_{ij}\left(x_{j}-\bar{x}\right)}{\sum_{i=1}^{n}{\left(x_{i}-\bar{x}\right)}^{2}/n}$$ (8)

In Equations (7) and (8), *n* denotes the count of spatial entities being analyzed; *x*_*i*_ and *x*_*j*_ denote the UGHQD levels of cities *i* and *j*, respectively; $\bar{x}$ denotes the mean value of UGHQD; and *W*_*ij*_ is the spatial weighting matrix.

#### 3.2.3 Cold and hot spots.

Although Moran’s I is primarily used to monitor the clustering of elements with similar values, it cannot identify statistically significant hot or cold spots. To further differentiate whether spatial autocorrelation characteristics represent “hot spots” (high values) or “cold spots” (low values), this study utilizes the *Getis-Ord G*_*i*_ * statistic method [61]. This method enables the examination of the clustering tendencies and distinct characteristics of UGHQD levels. The specific equation is provided below:


$$Z(G_{i}^{*})=\frac{G_{i}^{*}-E(G_{i}^{*})}{\sqrt{Var(G_{i}^{*})}}=\frac{\sum_{j=1}^{n}W_{ij}x_{j}-\sum_{j=1}^{n}x_{j}/n\sum_{j=1}^{n}W_{ij}}{\sqrt{\frac{\sum_{j=1}^{n}x_{j}^{2}}{n}-{\left(\bar{X}\right)}^{2}}\sqrt{\frac{[n\sum_{j=1}^{n}W_{ij}^{2}-(\sum_{\text{j=1}}^{n}W_{ij}{)}^{2}]}{n-1}}}$$ (9)

In this formula, *W*_*ij*_ represents the spatial weight matrix, *n* is the number of cities, and *x*_*j*_ denotes the UGHQD level of city *j*. *G*^ *^ value greater than E(G*) indicates the presence of a “hot spot” region, whereas a lower value signifies a “cold spot” region.

#### 3.2.4 Kernel density estimation.

To probe the distributional dynamics and spatial evolutionary trajectories of UGHQD levels more effectively, this study employs kernel density estimation. This method analyzes the location, evolution, and polarization of the distribution of UGHQD composite indexes both nationally and across the four major regions. We assume the density function of the UGHQD indicator is as follows:


$$f(y)=\frac{1}{Nh}\sum_{i=1}^{N}K\left(\frac{Y_{i}-y}{h}\right)$$ (10)

In this equation, *N* denotes the count of observations encompassed within the sample data set, which, for the present case, is 287. *Y*_*i*_ denotes the independent and identically distributed observations, y is the average value of these observations, and *h* is the bandwidth. For this analysis, we have selected the Gaussian Kernel density function, a technique prevalently adopted in scholarly research, to forecast the progressive changes of UGHQD metrics. The specific function is detailed in Equation (11).


$$k(y)=\frac{1}{\sqrt{2\pi }}\exp(-\frac{y^{2}}{2})$$ (11)

#### 3.2.5 BP neural network prediction model.

**Fig 2:**
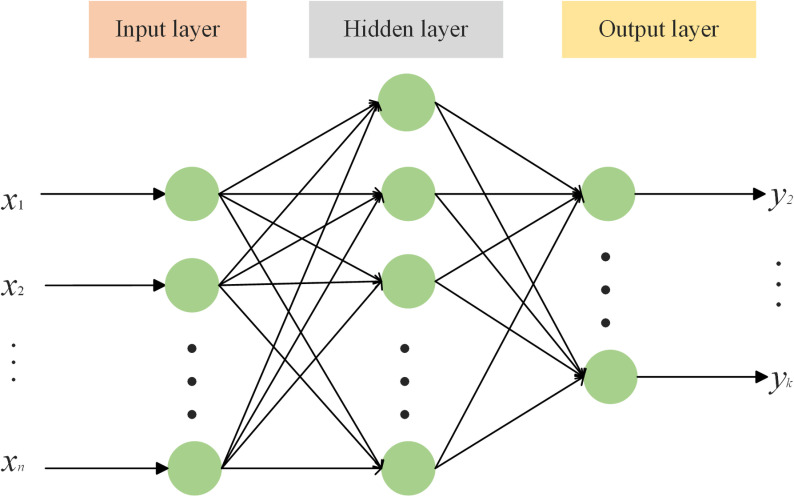
BP neural network prediction framework.

The BP (Back Propagation) neural network prediction model is an operational model with adaptive capabilities, designed to achieve arbitrary nonlinear mapping from inputs to outputs. It comprises input and output layers, along with one or more hidden layers. Signals are transmitted from the input nodes to the hidden layers and then to the output layer. Notably, each layer consists of several independent neurons. There is no interaction among neurons within the same layer; however, signal transmission occurs between neurons across different layers (Fig 2). Furthermore, unlike formal logical reasoning, the BP neural network employs signal forward propagation and error back propagation algorithms to analyze, summarize, simulate, and optimize historical data. Its adaptive capabilities enable it to predict developmental trends more accurately. To address the complex internal mechanisms of UGHQD, this study utilizes the BP neural network prediction model. After extensive iterative calculations, the final model configuration includes 11 neuron inputs, 1 hidden layer, and 5 neuron outputs.

#### 3.3 Data.

This study utilizes panel data from 2003 to 2020 as the research sample to examine the level of UGHQD across 287 cities in China. The primary data sources are the *China City Statistical Yearbook*, *China Urban Construction Statistical Yearbook*, and *China Science and Technology Statistical Yearbook*, along with statistical yearbooks from individual cities and the EPS database. Information on the quantity of green patents awarded from the State Intellectual Property Office of China’s patent exploration database (SIPO). For missing data, linear interpolation has been employed to compensate. Additionally, it is pertinent to note that the real GDP per capita data were calculated by deflating the GDP of each city to constant 2003 prices using the provincial GDP deflator and then dividing the result by the respective city’s total population count at the end of each calendar year.

## 4. Results

### 4.1 Level measurement results

**Fig 3:**
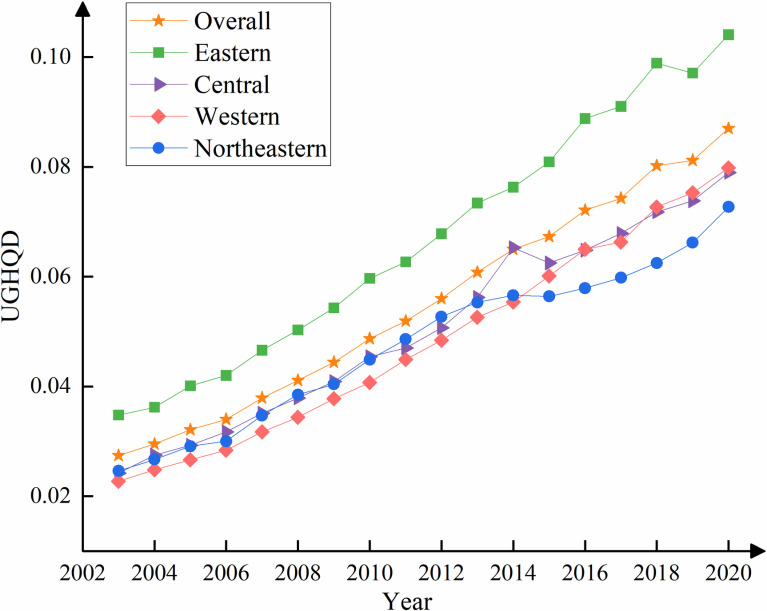
Trends in the level of UGHQD in China and four major regions.

In this analysis, the entropy method is used to assess the UGHQD levels across 287 cities in China. Fig 3 shows the progression of the mean UGHQD scores both nationally and across China’s four major regions from 2003 to 2020.

On the national scale, the average UGHQD score in China increased from 0.0256 in 2003 to 0.0855 in 2020, reflecting an average annual growth rate of 6.93%. This sustained positive trend is indicative of the widespread dissemination and adoption of green development principles across the country. In terms of public awareness, policy support, and practical implementation, there has been a significant and positive enhancement in environmental protection, leading to remarkable achievements.

In terms of the four major regions, the average UGHQD scores, ranked in descending order, are as follows: Eastern>  Central > Northeastern>  Western. Specifically, the eastern region experienced the most significant improvement, with its UGHQD score rising from 0.0348 in 2003 to 0.1041 in 2020, marking an increase of 199%. This consistent leadership can be attributed to the eastern cities’ notable advantages in geographical location, infrastructure, scientific and technological capabilities, industrial structure, and environmental protection. The UGHQD level in the Central region exhibited considerable fluctuations between 2013 and 2015, displaying an *N*-shaped trend of initial rise, subsequent decline, and a rebound. These fluctuations may reflect the impacts of policy adjustments, economic structural instability, and changes in the external economic environment during the study period. The western region, initially ranking the lowest until 2015, surpassed the northeast after that year, demonstrating a strong development momentum. These improvements can be attributed to policy support and increased investment, which have stimulated economic growth and environmental enhancement, thereby further driving the rapid advancement of UGHQD in the region. Meanwhile, the northeastern region’s score in UGHQD showed a fluctuating but overall upward trend, characterized by initial growth, a decline, and then a gradual recovery. The region’s long-term reliance on a heavy industrial and manufacturing-based economic structure, coupled with a relatively slow pace of economic transformation, has hindered its ability to effectively meet green development demands. Additionally, the Northeast’s limited innovation capacity and suboptimal policy implementation have further constrained its economic vitality and UGHQD potential.

### 4.2 Spatial distribution patterns

**Fig 4:**
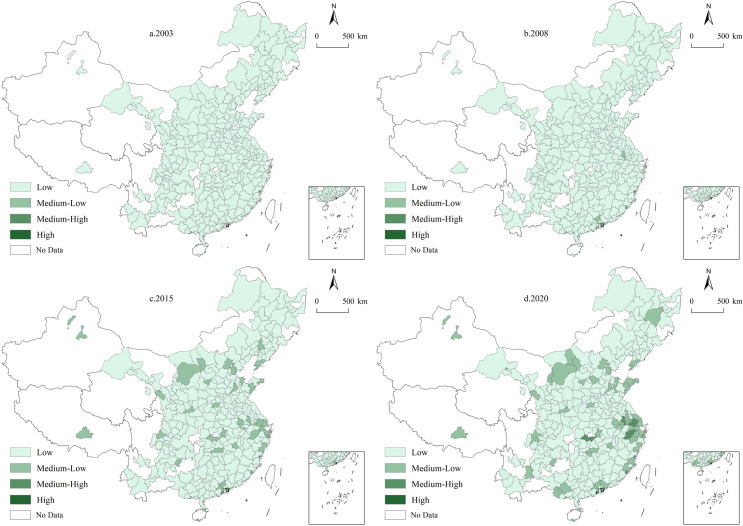
Spatial Evolution Trends of UGHQD level in China. Note: Quoted from the Tianditu map. Source of base map: the open source map data service provided by the National Platform for Common GeoSpatial Information Services (https://www.tianditu.gov.cn/).

**Fig 5:**
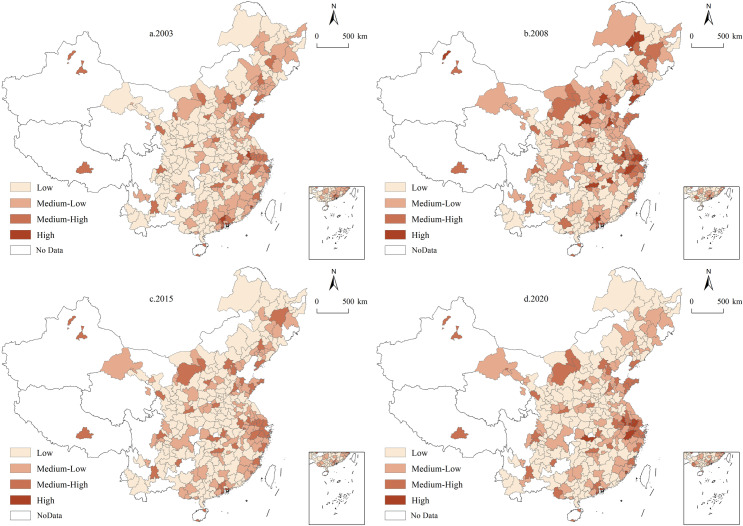
Spatial distribution of UGHQD level in China. Note: Quoted from the Tianditu map. Source of base map: the open source map data service provided by the National Platform for Common GeoSpatial Information Services (https://www.tianditu.gov.cn/).

This study utilized a comprehensive data set covering 287 cities across China from 2003 to 2020, using ArcGIS 10.8 software to examine the evolutionary trends and spatial distribution characteristics of UGHQD levels. The composite UGHQD index was categorized into four levels—low (0.01–0.10), medium-low (0.10–0.19), medium-high (0.19–0.28), and high (0.28–0.37)—using the equidistant spacing method to explore the spatial evolution trends (Fig 4). Additionally, to further illustrate the specific spatial distribution of UGHQD, a spatial visualization vector map was created based on the “natural breaks” classification method as described in existing studies [62–64] (Fig 5). Fig 5 illustrates the development levels at different time points, highlighting the disparate progression among cities.

According to Fig 4, UGHQD levels across 287 Chinese cities from 2003 to 2020 show a clear upward trend. During the study period, UGHQD levels gradually transitioned from lower to higher levels, with this change being particularly pronounced in the eastern region. Specifically, cities such as Hangzhou, Xiamen, Nanjing, Zhuhai, and Shenzhen in the east experienced a significant leap from low to medium-high and high UGHQD levels, with total growth rates exceeding 200%. This substantial improvement is primarily attributed to the dual drivers of economic policy support and technological advancement, which have effectively promoted economic transformation and upgrading, thereby significantly enhancing UGHQD levels in these regions. In contrast, UGHQD levels in the northeastern region remained relatively stable. Apart from some growth observed in Dalian, Harbin, and Shenyang, most cities in this region remained at low UGHQD levels throughout the study period. This stagnation is largely due to the aging industrial base and a lack of new economic drivers, making short-term improvements in UGHQD challenging. In the central and western regions, UGHQD levels mainly transitioned from low to medium-low levels, with central cities such as Beijing, Chengdu, Nanning, Zhengzhou, and Xi’an showing significant progress. By the end of the study period, UGHQD composite index values for these cities had approached medium-high levels, indicating potential for further advancement. In recent years, these regions have actively implemented national green development policies by adjusting economic structures and accelerating industrial upgrades, which has effectively promoted sustainable regional economic development and enhanced UGHQD levels.

As shown in Fig 5, the cities with high UGHQD levels in 2003, 2008, 2015, and 2020 were predominantly located in eastern coastal and economically significant cities, such as Shenzhen, Zhuhai, Hangzhou, Guangzhou, Beijing, and Shanghai. These cities, as core drivers of China’s economic growth, have achieved significant levels of UGHQD due to their strategic geographic locations, resource endowments, industrial strengths, and robust public service systems. Furthermore, the regional clustering effect reinforces their potential to sustain leadership in future development. Cities with medium-high and medium-low UGHQD levels, primarily located in the provinces of Henan, Anhui, Hebei, and Liaoning, are mainly situated in China’s central and northeastern regions, and their classification remains relatively stable. Among the 287 cities in the national sample, those with low levels of UGHQD, predominantly found in the central and western regions, constitute a notable proportion. In summary, the main spatial distribution characteristics of UGHQD in China can be characterized as “high in the east, moderate in the center, and low in the west”.

### 4.3 Spatial correlation analysis

#### 4.3.1 Global spatial autocorrelation.

To assess the spatial correlation of comprehensive UGHQD levels across 287 Chinese cities, this study calculates the Global Moran’s I for the period from 2003 to 2020 using Stata 17.0, employing both geographic adjacency and economic distance weight matrices (Table 2).

**Table 2:** Results of the calculation of Moran’s I.

Year	Geographic adjacency matrix		Economic distance matrix	
	Moran’s I	Z value	Moran’s I	Z value
2003	0.060	11.003	0.315	3.899
2004	0.059	10.183	0.292	3.378
2005	0.056	9.971	0.282	3.388
2006	0.057	10.117	0.307	3.646
2007	0.061	10.595	0.325	3.821
2008	0.061	10.598	0.371	4.348
2009	0.060	10.603	0.349	4.166
2010	0.053	9.464	0.269	3.218
2011	0.066	11.598	0.384	4.552
2012	0.067	11.649	0.361	4.271
2013	0.065	11.291	0.391	4.617
2014	0.067	11.630	0.430	5.021
2015	0.067	11.648	0.415	4.903
2016	0.073	12.858	0.484	5.761
2017	0.082	14.229	0.550	6.487
2018	0.086	14.985	0.567	6.715
2019	0.079	13.663	0.506	5.975
2020	0.087	14.890	0.599	6.996

As shown in Table 2, under the geographic adjacency weight matrix, Global Moran’s I is significantly positive, although with a low coefficient value. This suggests positive spatial autocorrelation in UGHQD levels, but the spatial clustering effect is not pronounced. In contrast, under the economic distance weight matrix, Moran’s I value of UGHQD levels is consistently and significantly positive, indicating a substantial positive spatial correlation in UGHQD across China. Notably, under both spatial weighting matrices, Moran’s I demonstrates a variable increase throughout the sample period, peaking in 2020. In absolute terms, Moran’s I value calculated using the economic distance matrix exceeds the value derived from the geographic adjacency matrix. The positive spatial spillover effect under economic distance conditions strengthens over time, suggesting that regions with similar economic conditions exhibit a more pronounced positive spatial spillover effect.

#### 4.3.2 Local spatial agglomeration patterns.

**Fig 6:**
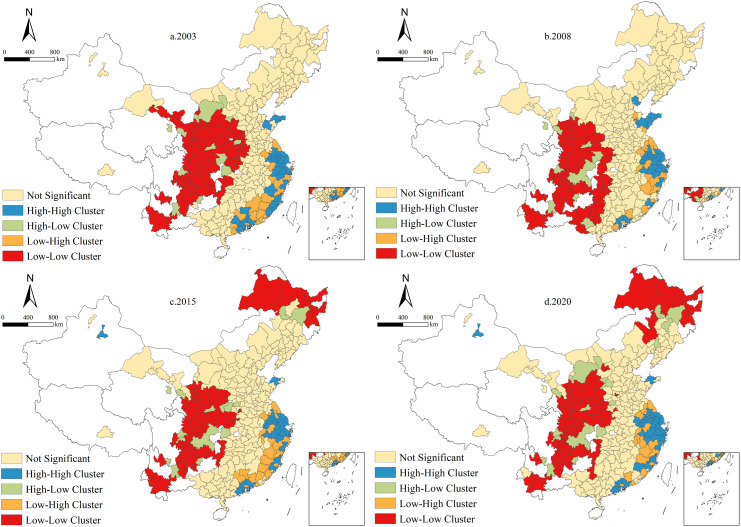
The LISA cluster diagram of UGHQD. Note: Quoted from the Tianditu map. Source of base map: the open source map data service provided by the National Platform for Common GeoSpatial Information Services (https://www.tianditu.gov.cn/).

To provide deeper insights into the local spatial association patterns of UGHQD, this study employs cross-sectional data from 2003, 2008, 2015, and 2020. Utilizing ArcGIS and GeoDa software, Local Indicators of Spatial Association (LISA) clustering maps are generated at a significance level of P < 0.05 (Fig 6). The spatial clustering of UGHQD levels across 287 Chinese cities is categorized into four types: “high-high (HH)”, “high-low (HL)”, “low-high (LH)”, and “low-low (LL)”. These categories facilitate the assessment of the agglomeration status and distribution characteristics of each city. Notably, “high-high” and “low-low” clustering indicates that an area with high (low) attribute values is surrounded by areas with similarly high (low) values, suggesting a positive spatial correlation. Conversely, “high-low” and “low-high” clustering occurs when a geographical entity characterized by high (or low) metrics is encircled by entities showcasing low (or high) metrics, indicating a negative spatial correlation.

As illustrated in Fig 6, the “high-high” and “low-high” clustering areas are predominantly located in the eastern region. Their spatial distribution remains relatively stable across the four selected years, with no significant fluctuations. In 2003 and 2008, “high-low” and “low-low” clustering areas were primarily found in the central and western regions. However, in 2015 and 2020, these clusters extended to include several cities in the northeastern region as well. Overall, the local agglomeration patterns of UGHQD in China’s 287 cities have exhibited minimal change, demonstrating a certain degree of spatio-temporal inertia.

#### 4.3.3 Local spatial cold and hot spot analysis.

**Fig 7:**
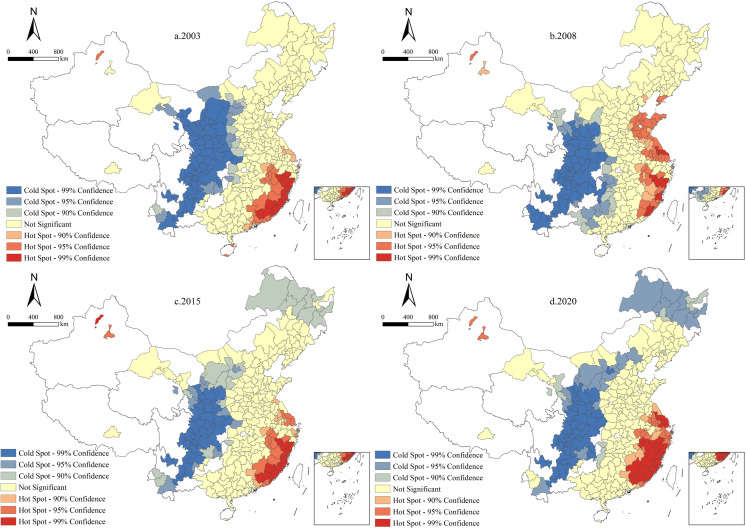
Cold and hot spot evolution of UGHQD. Note: Quoted from the Tianditu map. Source of base map: the open source map data service provided by the National Platform for Common GeoSpatial Information Services (https://www.tianditu.gov.cn/**).**

To identify whether a city falls within a hot or cold spot of UGHQD, this study employs ArcGIS 10.8 to compute the Local Getis-Ord G* index values for 287 Chinese cities. Based on these values, spatial distribution areas are classified into hot spots, cold spots, and areas of random distribution. Specifically, hot (cold) spots are categorized into three levels: significant hot (cold) spots (99% confidence interval), moderate hot (cold) spots (95% confidence interval), and mild hot (cold) spots (90% confidence interval), as shown in Fig 7.

As shown in Fig 7, an analysis of the years 2003, 2008, 2015, and 2020 reveals that high-cold-spot areas are predominantly found in the central and western parts of the country, while moderate and low-cold-spot areas are primarily situated around these high-cold-spot regions and in the northeastern region. Compared to other areas with non-random distributions, the cold spot regions encompass a significantly larger area, thereby unveiling the spatial differentiation pattern of UGHQD. The diverse topography of the central and western regions, including mountains, plains, basins, and rivers, predominantly feature extensive plains and basins. These terrains are highly conducive to human settlement and agriculture, leading to an increase in human activity and a subsequent expansion of non-ecological environments, which negatively impact UGHQD levels. As a result, plains and basins often exhibit a high cold-spot phenomenon, which is detrimental to ecological environment protection. Conversely, areas with a high density of hot spots are predominantly found in the eastern region, with moderate and low hot-spot areas falling within the influence radius of these high hot-spot areas. This distribution is attributed to the high hot-spot areas’ superior resource endowments, large market scale, and excellent infrastructure, creating a potent economic driving force. The interconnected development of cities within high, moderate, and low hot spot areas generally fosters a favorable UGHQD level, predominantly reflecting hot spot distribution characteristics. Overall, the spatial distribution of UGHQD exhibits a pattern of “hot in the east and cold in the west”.

### 4.4 Spatial orientation characterization

**Fig 8:**
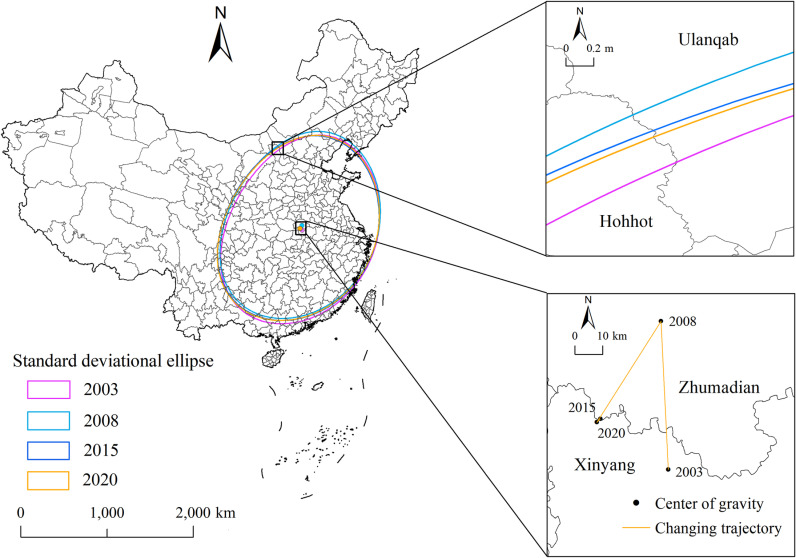
The standard deviation ellipse of UGHQD. Note: Quoted from the Tianditu map. Source of base map: the open source map data service provided by the National Platform for Common GeoSpatial Information Services (https://www.tianditu.gov.cn/).

To quantitatively determine the spatial evolution trends of UGHQD across 287 Chinese cities, this study selects 2003, 2008, 2015, and 2020 as representative periods. The standard deviation ellipse method at the first standard deviation level is used to derive relevant parameters (Table 3), which visually depict the scale distribution and spatio-temporal evolution characteristics of UGHQD. Furthermore, leveraging the center of gravity model and ArcGIS 10.8, the standard deviation ellipse of UGHQD and the trajectory of the center of gravity are generated (Fig 8).

**Table 3:** Standard deviation ellipse parameters of UGHQD.

Year	Deflection	Oblateness	Barycentric coordinate	Perimeter (km)	Area (km2)
2003	51.844°	0.247	(114.212°, 32.327°)	61.778	2947184
2008	54.230°	0.238	(114.238°, 32.799°)	61.901	2966386
2015	56.175°	0.224	(113.953°, 32.497°)	61.683	2956152
2020	56.003°	0.236	(113.968°, 32.507°)	61.836	2961319

As shown in Table 3, the area of the standard deviation ellipse in 2020 exceeds that of 2003, though the difference is marginal. This suggests that the distribution range of UGHQD across cities, relative to their average, is more dispersed, with some outliers present, yet without significant differences among cities. The spatial distribution exhibits a slight trend of expansion. Additionally, the changes in the standard deviation ellipse’s orientation and centroid coordinates for UGHQD in 2003, 2008, 2015, and 2020 are minimal, and the ellipse’s flattening ratio indicates a slight narrowing trend, suggesting an increase in the directionality of UGHQD. According to Fig 6, the mean geometric centroid coordinates of the standard deviation ellipse are approximately (114°09′03″E, 32°53′25″N), primarily located in Henan Province. However, considering the higher level of UGHQD in the eastern coastal cities compared to those in the centroid region, a non-normal spatial distribution exists. The trajectory of the center of gravity moved from within Xinyang city in 2003 to northward of Zhumadian city in 2008 and then migrated from north to south to the junction of these two cities in 2015 and 2020. This movement pattern demonstrates an evolutionary characteristic from east to west, south to north, and back south. The declination angle varied from 51.844° to 56.175°, averaging 54.573°, and the overall spatial distribution pattern was “northeast-southwest”. Specifically, the declination angle increased from 51.844° in 2003 to 56.175° in 2015, indicating a strengthening “northeast-southwest” distribution pattern of UGHQD, and slightly decreased to 56.003° in 2020, suggesting a slight weakening of this pattern.

### 4.5 Dynamic evolution analysis

**Fig 9:**
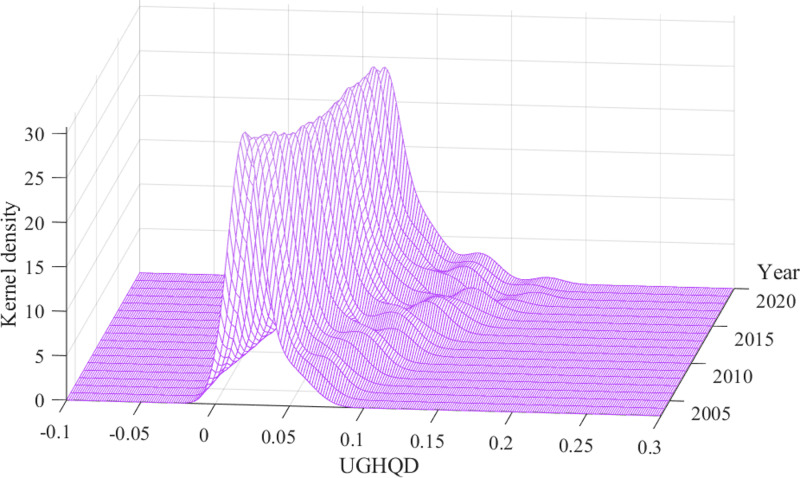
Distribution dynamics of UGHQD in China.

**Fig 10:**
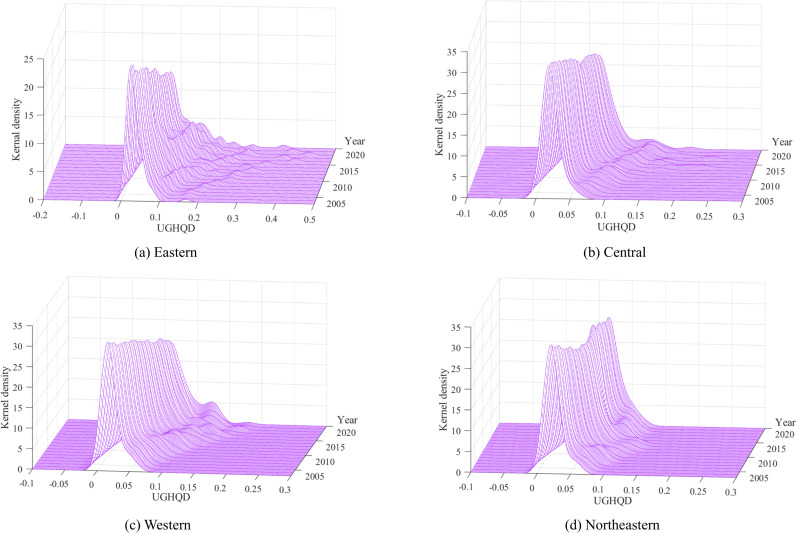
Distribution dynamics of UGHQD in the four major regions.

To comprehensively understand spatio-temporal dynamics in the distribution of UGHQD levels across China, this study employs the Kernel density estimation. This approach facilitates a comprehensive depiction of the evolution characteristics of UGHQD’s distribution in China throughout the observation period. Fig 9 presents the Kernel density estimation of UGHQD levels throughout China, while Fig 10 displays the Kernel density estimation of cities within the four major regions. A detailed analysis is provided, examining the distribution curves from the perspectives of location, shape, flexibility, and polarization features.

Regarding the distribution location, the distribution curves for UGHQD levels in China and its four major regions exhibit rightward shifts to varying degrees, indicating a significant overall increase in UGHQD. This trend aligns with the objective observations detailed earlier. Notably, during the study period, the eastern region displayed a more pronounced rightward shift of its main peak compared to other regions, indicating a relatively rapid enhancement in the UGHQD level in this region. At the forefront of China’s economic development, the eastern region has experienced significantly higher economic growth compared to other areas. This growth has fueled greater investment in green infrastructure and technological innovation. Leveraging its policy advantages, the eastern region has prioritized green development strategies, optimized resource allocation, and accelerated the green transformation of industries, leading to a substantial increase in UGHQD levels. In contrast, other regions have shown less pronounced shifts in their peaks, primarily due to weaker industrial foundations and slower progress in green transformation.

Regarding the distribution shape, the peaks of the UGHQD distribution curve throughout the observation period generally exhibit a “V-shaped” trend. The main peak’s height fluctuates upward, and its shape evolves to become “sharper and narrower” over time. This evolution signifies an increasingly pronounced trend towards a centralized distribution of UGHQD across China, with the absolute regional disparities gradually narrowing. This trend can be attributed to the accelerated pace of green transformation within the region, coupled with strengthened inter-regional synergies and cooperation. These factors not only promote the rational allocation of resources but also contribute to reducing regional development imbalances and enhancing UGHQD levels nationwide. Regionally, the main peak of the distribution curve for the level of UGHQD within the eastern and western regions displays a “fluctuating downward” trend, suggesting an increasing divergence in UGHQD levels between these regions over time. Although the eastern region benefits from well-developed green infrastructure and strong policy support, these resources and initiatives are predominantly concentrated in a few central cities. This concentration limits the opportunities for UGHQD in peripheral cities, thereby exacerbating development imbalances within the region. Similarly, in the western region, economically advanced cities have leveraged favorable policies to accelerate their UGHQD progress, while less developed areas have lagged. This internal disparity has further widened regional differences in UGHQD levels. In the central region, the peak’s value follows an “inverted U-shaped” trend, suggesting that UGHQD in the central region initially becomes more concentrated before dispersing throughout the observation period. In the early stages of the green transition, concentrated policy support and financial investment significantly boosted UGHQD levels in central cities. Over time, the benefits of this improvement gradually spread to other cities within the region, helping to reduce intra-regional disparities. The main peak’s height in the northeastern region exhibits significant fluctuations during the sample period, transitioning from relatively broad and flat to narrow and steep. This transition illustrates the spatial pattern of UGHQD levels evolving from dispersed to more centralized. Over time, the concentration of UGHQD in a few central cities in the Northeast has intensified, while other cities in the region have fallen behind, resulting in a gradual widening of intra-regional disparities.

Regarding the distribution flexibility, the distribution curves of UGHQD in China and its four major regions all exhibit a “right-tail” phenomenon to varying extents. Nationally, the overall “right-tail” trend of UGHQD levels in Chinese cities has been gradually lengthening over the years, although the rate of change is minimizing. This suggests that while regional disparities in UGHQD levels persist, they are not markedly pronounced. From a regional perspective, all four major areas show a “right-tail” distribution curve for UGHQD levels, indicating that certain cities within these regions demonstrate significantly higher levels of UGHQD. Specifically, the “right-tail” phenomenon is more pronounced in the eastern and central regions, resulting in a broader distribution spread. This is attributed to the relatively higher UGHQD levels of central cities in these regions, which further accentuates the inter-city disparities. The western region’s UGHQD pattern is characterized by an initial broadening followed by convergence, whereas the northeastern region exhibits a trend toward gradual convergence over time. This indicates that the UGHQD levels in cities within the northeastern and western regions have been relatively stable, with the gap between cities narrowing progressively. In general, resources and policies tend to favor central cities with strong economic foundations and well-developed infrastructures, granting these cities a first-mover advantage in UGHQD development. This can lead to an unbalanced development trend within the region. To effectively address this issue, the government should formulate differentiated green development policies tailored to the specific economic conditions and infrastructure needs of various regions, ensuring that less developed areas receive the necessary resources and support. Additionally, establishing regional cooperation mechanisms to promote inter-regional information sharing and resource mutual assistance, particularly in green technology and sustainable practices, can facilitate shared progress across regions. This approach would help narrow disparities in UGHQD levels, promote balanced development nationwide, and contribute to the comprehensive sustainable development of both the economy and the environment.

Concerning the distribution polarization, the national distribution of UGHQD levels exhibits a degree of polarization, yet the lower peak values of side peaks indicate a mild degree of polarization. Further analysis reveals that in the four major regions, the distribution is characterized as “a single primary peak with multiple secondary peaks”, evolving towards a multipolar trend. Specifically, the Kernel density distribution curve in the eastern region features one main peak and several secondary peaks, with a distinct gradient effect between the primary and secondary peaks, reflecting the hierarchical nature of UGHQD in this area. In the central region, the notable difference in elevation between the primary and secondary peaks in the Kernel density distribution curve suggests a subtle trend towards a multipolar distribution of UGHQD levels. The western region’s Kernel density distribution curve transitioned from having no secondary peaks in 2009 to one, and then to two after 2018, establishing a multipolar trend with one primary peak and two secondary peaks. The northeastern region’s Kernel density distribution curve exhibits an increase and subsequent decrease in the number of secondary peaks, ending with a single primary peak in the sample’s end, indicating a gradual mitigation and control of polarization phenomena. Overall, the level of UGHQD in China and its four major regions shows a clear trend of divergence. This divergence may stem from significant disparities in the efficiency and effectiveness with which cities implement environmental policies. Non-central cities often face greater challenges in advancing green development due to resource constraints, poor management, or lack of technical support, directly impacting their UGHQD levels. Additionally, public environmental awareness and participation play a crucial role in shaping UGHQD levels. In regions where environmental awareness is relatively low, policy support alone is insufficient without active public engagement, making effective policy implementation difficult and exacerbating regional disparities. To address this, the government should enhance the supervision and evaluation of environmental policy implementation, while also increasing public awareness and participation in green lifestyles through education and public campaigns. Such efforts would help gradually reduce the disparities in UGHQD levels between cities and promote a more balanced state of green quality development nationwide.

### 4.6. Forecast of UGHQD levels

**Fig 11:**
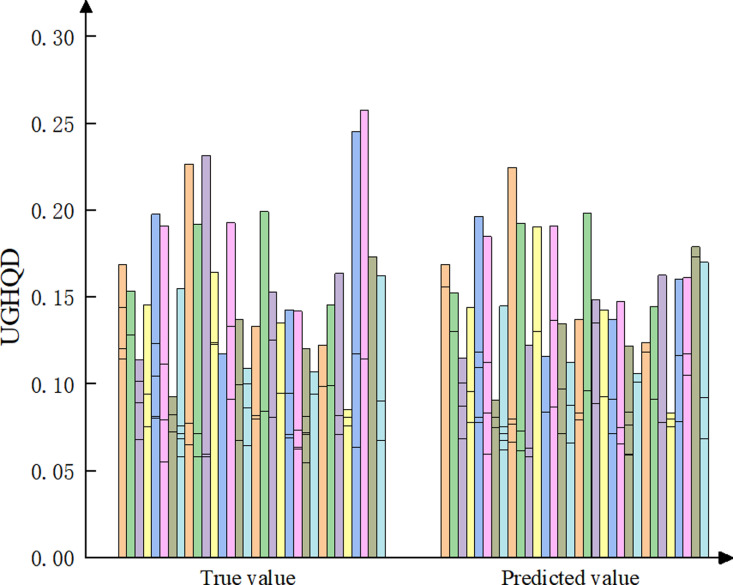
Predicted and true value results.

This study projects the future trends of UGHQD levels across Chinese cities, based on their assessment and dynamic evolution analysis, to develop effective strategies for enhancing UGHQD. These strategies aim to optimize urban development patterns, improve environmental quality, and foster economic prosperity. Specifically, the BP neural network model was developed using Matlab 2022b, performing iterative calculations to forecast the average UGHQD index for China and its four major regions—East, Center, West, and Northeast—for the period 2021–2025 (Table 4). By employing the actual UGHQD index values from 287 Chinese cities in 2020 as test data, it was found that the absolute value of the relative error between the actual and forecasted values is less than 5%. This suggests that the BP neural network model demonstrates a good fit, high accuracy, and a reliable level of confidence for future projections. Fig 11 visually illustrates that the overall difference between the forecasted and actual UGHQD values in 2020 is minor, though some cities show significant fluctuations. This variability is attributed to the comprehensive economic impact of the COVID-19 pandemic’s onset and global spread in 2020. Given that the BP neural network forecasting model primarily relies on historical data, it may not accurately capture the effects of such unforeseen events, resulting in notable discrepancies between predicted and actual values for some cities in 2020. However, with the Chinese economy showing signs of recovery from 2021 onwards, forecasting UGHQD from 2021 to 2025 has significant practical relevance.

**Table 4:** Predicted results of UGHQD.

Regions	2020			2021	2022	2023	2024	2025
	True value	Predicted value	Relative error					
Overall	0.0870	0.0860	0.0111	0.0918	0.0973	0.1041	0.1108	0.1160
Eastern	0.1041	0.0999	0.0396	0.1035	0.1061	0.1124	0.1176	0.1211
Central	0.0790	0.0794	0.0056	0.0869	0.0927	0.0987	0.1053	0.1088

Table 4 suggests that the UGHQD levels in Chinese cities are projected to continue their upward trajectory. During the 2021-2025 forecast period, most cities in the eastern region are expected to maintain their leading position in UGHQD. Notably, a few cities in the western region are on track to reach high levels of development, which could catalyze development in surrounding cities. Overall, these regions possess significant potential for UGHQD, with pathways to achieving such development being both feasible and sustainable. In contrast, under the existing development paradigm, the expected advancement of UGHQD within the northeastern and central regions appears limited. Addressing the existing development issues, optimizing green innovation pathways, and breaking through barriers to higher UGHQD levels are urgently required.

## 5 . Discussion

Given the escalating severity of ecological challenges, the coordinated advancement and integration of economic development, social progress, and ecological civilization are key drivers of UGHQD. These elements not only motivate and support each other but also together form a strategic blueprint for the sustainable urban development. At a pivotal moment of economic and social transition, investigating the regional variations in UGHQD and its evolving trends is of paramount importance. Against this backdrop, this study conducts a systematic empirical investigation into the development levels, dynamic trends, and future forecasts of UGHQD, grounded on a theoretical understanding of its essence. The findings delineate that the eastern region surpasses others in UGHQD levels, attributed to its robust economic base, outstanding innovation ecosystem, abundant resources, and effective policy support [18]. Exploratory spatial correlation analysis reveals a positive spatial autocorrelation in UGHQD levels, with pronounced clustering. This is particularly evident in the eastern region, where the spatial agglomeration of cities with advanced green development is most distinct. This phenomenon largely results from an amalgamation of factors such as geographical advantages, pioneering initiatives, talent aggregation, and policy backing. As China’s most economically formidable and promising region, the eastern region plays a critical role in championing UGHQD.

Our empirical findings further reveal varying degrees of polarization in UGHQD levels across China and its four major regions. Additionally, although the UGHQD distribution curve shows a “right-tail” trend, the probability of extreme values decreases over time, indicating a reduction in disparity expansion among cities [65]. This moderation is likely influenced by the positive impacts of regional integration policies. The focus of the Chinese government in recent years on promoting resource sharing, connectivity, and synergistic development among different regions, has effectively mitigated the scattered distribution of UGHQD levels among cities within these regions. BP neural network predictive analysis indicates an increasing probability for cities at lower levels of UGHQD to transition to higher levels, signifying a future enhancement in China’s overall UGHQD. To attain high-level development objectives, the government has been promoting green and sustainable urban development through policies focused on environmental protection, resource conservation, and low-carbon growth [66, 67]. Concurrently, elevating the level of technological innovation enhances resource utilization efficiency in cities, reduces environmental pollution, and fosters green lifestyles and environmental stewardship, collectively propelling UGHQD toward greater heights.

This study offers potential contributions in two significant areas: Firstly, it broadens the empirical investigation to the city level, thereby extending past the focus on provincial data predominantly examined in the existing literature. This expansion enables a more precise and granular empirical analysis. Utilizing data from 287 cities spanning 2003 to 2020, this study provides a thorough examination of the spatial distribution patterns and dynamic evolution trends of UGHQD. Such analysis aids in more accurately identifying strategies and focal points for future enhancement of UGHQD, offering empirical support for the scientific development of related policies. Secondly, this study goes beyond existing studies that primarily use GTFP as a metric, by constructing a comprehensive evaluation framework for UGHQD. This framework, grounded in the three dimensions of economic development, social progress, and ecological civilization, aligns more closely with the fundamental goals of UGHQD. It is through this nuanced level of evaluation that we can more clearly identify the current shortcomings and challenges in the UGHQD of China. Nonetheless, this study is not without its limitations, such as the lack of in-depth exploration into the driving factors of UGHQD. We anticipate future research will offer a more systematic and comprehensive investigation into the underlying drivers of UGHQD, thereby providing more scientifically robust and precise policy recommendations.

## 6. Conclusions

This study evaluates UGHQD levels across 287 cities in China from 2003 to 2020. Drawing on the understanding of the concept of UGHQD, we developed a comprehensive evaluation indicator system encompassing the dimensions of economic development, social progress, and ecological civilization. Utilizing the entropy method, we assessed the UGHQD levels of these cities and explored their spatial distribution patterns using ArcGIS 10.8 software. Furthermore, we employed Moran’s I, cold and hot spots analysis, standard deviation ellipse, Kernel density estimation, and a BP neural network prediction model, we analyzed the spatio-temporal dynamics and forecasted future trends of UGHQD both nationally and in the four major regions. Our findings reveal: (1) A stable upward trend in UGHQD levels nationwide and across all four major regions, with the eastern region particularly standing out for its superior development. (2) A spatial imbalance in UGHQD levels, characterized by a concentration of the spatial distribution’s center of gravity in key cities and the eastern coastal areas. (3) Varying degrees of divergence in UGHQD throughout China and its four major regions, with the eastern region showing the most significant divergence. (4) Continued growth in UGHQD levels across China and its four major regions, with the eastern region expected to maintain its lead and exert a radiative effect on the development of other regions.

Based on the findings of this study, the following policy recommendations are proposed:

(1) Eliminate regional barriers to foster coordinated UGHQD across regions. As the leader in UGHQD, the eastern region not only provides robust momentum within its territory but also facilitates synchronized progress with other regions through its capacity to radiate influence. All four major regions must leverage this momentum, capitalize on their unique strengths, enhance collaboration in pivotal sectors such as economic expansion, societal progress, and environmental sustainability, and collectively forge a new era of cohesive UGHQD in China.
(2) Intensify the enforcement and evaluation of policies of UGHQD, while embracing green production and living practices. Embracing the principle that “lucid waters and lush mountains are invaluable assets”, we advocate for the sustainable utilization of resources, waste reduction, and productivity enhancement. Cities should expedite the green transition and modernization of industries to consolidate gains in energy efficiency and reduce emissions. This endeavor requires a collaborative approach to align economic and social development with ecological sustainability, aiming to fulfill the vision of UGHQD.
(3) Establish an interregional platform for green technology innovation to nurture and expand green industries. Focusing on green innovation, we should concentrate on fostering advanced green industries, supporting the growth of emerging sectors such as renewable energy and new materials, thus generating new green momentum. Furthermore, the creation of a green technology innovation-sharing system is crucial to transform the “siphon effect” into a “radiation effect”, stimulating the progress of less developed neighboring cities, diminishing regional development disparities, and ultimately elevating the comprehensive development standard of the entire region.

## Supporting information

S1 Data Corresponding data of methods. (ZIP)
